# Variation in Care for Patients with Irritable Bowel Syndrome in the United States

**DOI:** 10.1371/journal.pone.0154258

**Published:** 2016-04-26

**Authors:** Brian E. Lacy, Haridarshan Patel, Annie Guérin, Katherine Dea, Justin L. Scopel, Reza Alaghband, Eric Qiong Wu, Reema Mody

**Affiliations:** 1 Division of Gastroenterology & Hepatology, Dartmouth-Hitchcock Medical Center, Lebanon, New Hampshire, United States of America; 2 Immensity Consulting, Inc., Chicago, Illinois, United States of America; 3 Analysis Group, Inc., Montreal, QC, Canada; 4 US Medical Affairs, Takeda Pharmaceuticals International, Inc., Deerfield, Illinois, United States of America; 5 Analysis Group, Inc., Boston, Massachusetts, United States of America; 6 Global Outcomes and Epidemiology Research, Takeda Pharmaceuticals International, Inc., Deerfield, Illinois, United States of America; Charité-Universitätsmedizin Berlin, Campus Benjamin Franklin, GERMANY

## Abstract

**Objectives:**

Irritable bowel syndrome (IBS) affects nearly one in seven Americans. Significant national variations in care may exist, due to a current lack of standardized diagnosis and treatment algorithms; this can translate into a substantial additional economic burden. The study examines healthcare resource utilization in patients with IBS and in the subset of IBS patients with constipation (IBS-C) and analyzes the variation of IBS care for these patients across the United States (US).

**Methods:**

Healthcare resource use (HRU), including gastrointestinal (GI) procedures and tests, all-cause and intestinal-related medical visits, GI specialist visits, and constipation or diarrhea pharmacy prescriptions for IBS patients enrolled in a large US administrative claims database (2001–2012) were analyzed for the 24-month period surrounding first diagnosis. Multivariate regression models, adjusting for age, gender, year of first diagnosis, insurance type, and Charlson comorbidity index, compared HRU across states (each state vs. the average of all other states).

**Results:**

Of 201,322 IBS patients included, 77.2% were female. Mean age was 49.4 years. One in three patients had ≥3 distinct GI medical procedures or diagnostic tests; 50.1% visited a GI specialist. Significant HRU differences were observed in individual states compared to the national average. IBS-C patients had more medical visits, procedures, and pharmacy prescriptions for constipation/diarrhea than IBS patients without constipation.

**Conclusions:**

This study is the first to identify considerable regional variations in IBS healthcare across the US and to note a markedly higher HRU by IBS-C patients than by IBS patients without constipation. Identifying the reasons for these variations may improve quality of care and reduce the economic burden of IBS.

## Introduction

Irritable bowel syndrome (IBS) is a common medical condition with a prevalence of 9% to 14% in the general population [1]. It impairs patients’ quality of life and has a detrimental impact on the healthcare system [2–4]. Clinically, IBS is characterized by the presence of abdominal pain or discomfort associated with disordered defecation. According to the Rome III guidelines, IBS can be classified into 3 main types, based on predominant bowel habits: IBS with constipation (IBS-C), IBS with diarrhea (IBS-D), and IBS with alternating constipation and diarrhea (IBS-M) [5]. Although IBS is chronic and common, diagnosing and adequately treating it can be challenging for many healthcare providers due to its non-specific symptoms, the presence of overlapping upper and lower abdominal symptoms (e.g., reflux, dyspepsia, pelvic floor dysfunction), and the presence of co-existing somatic and psychological disorders [1,5]. IBS is also a heterogeneous disorder; patients with similar symptoms have highly variable responses to therapeutic interventions [6,7]. These barriers to effective care have led to the release of clinical guidelines to help improve the diagnosis and treatment of IBS [8]–specifically, by standardizing the diagnosis and treatment of specified conditions and thus minimizing existing variations in health care. This is an important step, because such variations can lead to inappropriate or repeated diagnostic tests and ineffective or unnecessary costly treatments [9], both of which further burden the healthcare system.

Regional variation in care and practice has been studied in a variety of disease areas, including cardiovascular and cerebrovascular diseases [10–16], orthopedic surgery [17], breast [18], colon [19], and prostate cancer [20], nephrology [21,22] and epilepsy [23]. Although not often studied in the field of gastroenterology, analyzing variations in health care is not a new concept. One study conducted in a population of patients with inflammatory bowel disease demonstrated substantial geographic variations in the use of biologics and hospitalization with and without surgery [24]. Another study analyzed the regional variation in the epidemiology of appendicitis and appendectomy in United States [25]. However, to our knowledge, the variation of care across the United States (US) has not been studied among patients with IBS.

The objectives of this study were to determine whether variations in health care for IBS patients exist among different states in the US and between different IBS subtypes. Given the known economic and clinical burden associated with constipation [26–30], and the lack of information on how care may be affected by the presence of constipation in patients with IBS, the second objective of this study focused on subgroups of patients with IBS-C and IBS patients without constipation.

## Materials and Methods

### Data source

This retrospective cohort study used data from the Truven Health Analytics MarketScan® Databases, a private-sector data source of enrollees covered by health benefit programs of large employers (>130 different insurance companies). The data represent the medical claims of insured employees and their dependents, as well as Medicare-eligible retirees with employer-provided Medicare supplemental plans. All census regions are represented, although there is a slightly higher representation from the South and North Central (Midwest) regions [31]. The MarketScan Research Databases are de-identified and are fully compliant with the Health Insurance Portability and Accountability Act of 1996 (HIPAA). Because this study did not involve the collection, use, or transmittal of individually identifiable data, Institutional Review Board review or approval was not required (retrospective studies based on de-identified data require no individual IRB approval).

### Patient selection

Patients were included in the study if they 1) had at least two diagnoses for IBS (International Classification of Diseases, 9th Revision [ICD-9] codes 564.1x) recorded on separate dates between January 1, 2001 and December 31, 2012; 2) had at least 12 months of continuous healthcare plan enrollment both before and after the first recorded IBS diagnosis; and 3) were at least 18 years of age on the date of the first recorded IBS diagnosis, with no upper age limit. Patients meeting these criteria were categorized by state of residence on the date of the first recorded IBS diagnosis. The sample was further divided into the subgroups of 1) patients with IBS-C (patients with at least one medical encounter associated with a diagnosis for constipation [ICD-9 code 564.0x] during the 24-month study period) and 2) IBS patients without constipation (patients without any diagnoses for constipation during the 24-month study period).

### Study period

The study period was defined as the 24-month period surrounding the first IBS diagnosis recorded in the database, i.e. 12 months before and 12 months after that date. All patients had medical and pharmacy claims information available during the entire study period.

### Measures and outcomes

Healthcare resource utilization (HRU) during the study period included gastrointestinal (GI) medical procedures and diagnostic tests, as well as medical visits (inpatient [IP] admissions, IP days, emergency room [ER] visits, office visits, other outpatient visits, other medical visits, and GI specialist visits) and pharmacy prescriptions for treating constipation or diarrhea. Among all the HRU components analyzed, the results of regional variations are presented on a subset of these components due to their clinical relevance as follows: 1) GI medical procedures and diagnostic tests, including colonoscopy; abdominal, colon, and pelvic computed tomography (CT) scan, non-therapeutic abdominal and pelvic ultrasound, and anorectal function testing (and proportions of patients with ≥3 such procedures/tests); 2) pharmacy prescriptions for treating constipation or diarrhea (see S1 Table for a list of all included prescription medications); and 3) medical visits, including intestinal-related (identified based on diagnosis codes for intestinal disorders [ICD-9 codes 560.xx-569.xx]) IP admissions and ER visits, and GI specialist visits.

### Statistical analyses

Regional variation in IBS care was analyzed by individually comparing the HRU rate in each state with the HRU rate in the rest of the US. Regional variation in care was analyzed using generalized linear regression models (GLM) with a log link and a negative binomial distribution for the analysis of the number of events. Regression models were adjusted for potential confounding factors, including age at the first IBS diagnosis, gender, type of healthcare plan, year of the first IBS diagnosis, and the modified Charlson Comorbidity Index (CCI) measured over the 24-month study period. Results were reported as adjusted incidence rate ratios (IRRs) with 95% confidence intervals (CIs) [32]. For the analysis of patients with three or more distinct GI medical procedures or diagnostic tests, the likelihood of having three or more such procedures or tests during the study period was analyzed using logistic regression models, and results from the comparisons were reported as adjusted odds ratios (ORs) with 95% CIs.

Variation in care between IBS patients without constipation and IBS-C patients was analyzed using a statistical approach similar the one described above, but instead of conducting statistical comparisons across states of residence, they were conducted between IBS-C patients and IBS patients without constipation, using the latter as the reference group.

## Results

A total of 201,322 IBS patients met the selection criteria, of whom 35,627 (17.7%) were categorized as IBS-C patients (Table 1). The mean patient age was 49.4 years and 77.2% were female; their average CCI was 1.6. The most common IBS-related comorbidities were abdominal pain (61.4%), gastroesophageal reflux disease (28.8%), headache (23.1%), and lower back pain (21.6%).

**Table 1:** IBS patient characteristics.

	IBS Patients
	N = 201,322
**Demographics**	
Age, Mean ± SD [Median]^a^	49.4 ± 15.4 [50.0]
Female, n (%)	155,449 (77.2)
**Region, n (%)**	
South	84,428 (41.9)
North-Central	53,666 (26.7)
West	37,784 (18.8)
North-East	25,444 (12.6)
**Insurance Plan Type, n (%)**	
Preferred Provider Organization (PPO)	116,160 (57.7)
Health Maintenance Organization (HMO)	30,302 (15.1)
Comprehensive Coverage	23,069 (11.5)
Point of Service (POS) / POS with Capitation	20,493 (10.2)
Consumer-directed Health Plan (CDHP)/ High-deductible HP (HDHP) or Exclusive Provider Organization (EPO)	7,134 (3.5)
Unknown ^b^	4,164 (2.1)
**Modified Charlson-Quan Comorbidity Index, Mean ± SD [Median]**^**c**^	1.6 ± 1.8 [1.0]
**IBS-Related Comorbidities**^**c**^**, n (%)**	
Abdominal Pain	123,653 (61.4)
Gastroesophageal Reflux Disease	57,962 (28.8)
Headache	46,523 (23.1)
Lower Back Pain	43,489 (21.6)
Constipation	35,627 (17.7)
Non-infectious Colitis	33,766 (16.8)
Diverticulosis	32,095 (15.9)
Chronic Pelvic Pain	24,572 (12.2)
Fibromyalgia	23,685 (11.8)
Asthma	21,957 (10.9)

Notes:

^a^ Age was calculated as of the first IBS diagnosis date.

^b^ "Unknown" includes patients from Puerto Rico and Virgin Islands as well as all those patients in the United States for whom geographic location information was not available.

^c^ Evaluated during the 24-month study period.

Abbreviations: IBS, irritable bowel syndrome; SD, standard deviation.

During the study period, colonoscopy was the most frequently conducted test; 44.9% of all patients underwent colonoscopy, with an average of 0.78 colonoscopies per patient (Table 2).

**Table 2:** Description of health care resource utilization in IBS patients.

	Patients with at least One Event, N (%)	Number of Events Mean ± SD [median]
**Medical Procedures and Diagnostic Tests**		
Colonoscopy	90,329 (44.9)	0.78 ± 1.08 [0.00]
CT Scan	69,570 (34.6)	1.33 ± 2.84 [0.00]
Ultrasound	70,874 (35.2)	0.72 ± 1.37 [0.00]
Anorectal Function Testing	5,345 (2.7)	0.05 ± 0.44 [0.00]
≥3 Distinct GI Medical Procedures or Diagnostic Tests	73,174 (36.4%)	-
**Pharmacy prescriptions for treating constipation or diarrhea**	67,706 (33.6)	0.76 ± 2.84 [0.00]
Treatment for Constipation	58,086 (28.9%)	0.59 ± 2.54 [0.00]
Treatment for Diarrhea	15,027 (7.5%)	0.17 ± 1.33 [0.00]
**Medical Visits**		
Intestinal-Related IP Admissions	14,701 (7.3%)	0.09 ± 0.39 [0.00]
Intestinal-Related ER Visits	13,729 (6.8%)	0.09 ± 0.40 [0.00]
GI Specialist Visits	100,940 (50.1%)	1.89 ± 2.85 [1.00]

Abbreviations: CT: computed tomography IP: inpatient; ER: emergency room; GI: gastrointestinal; SD: standard deviation.

**Fig 1:**
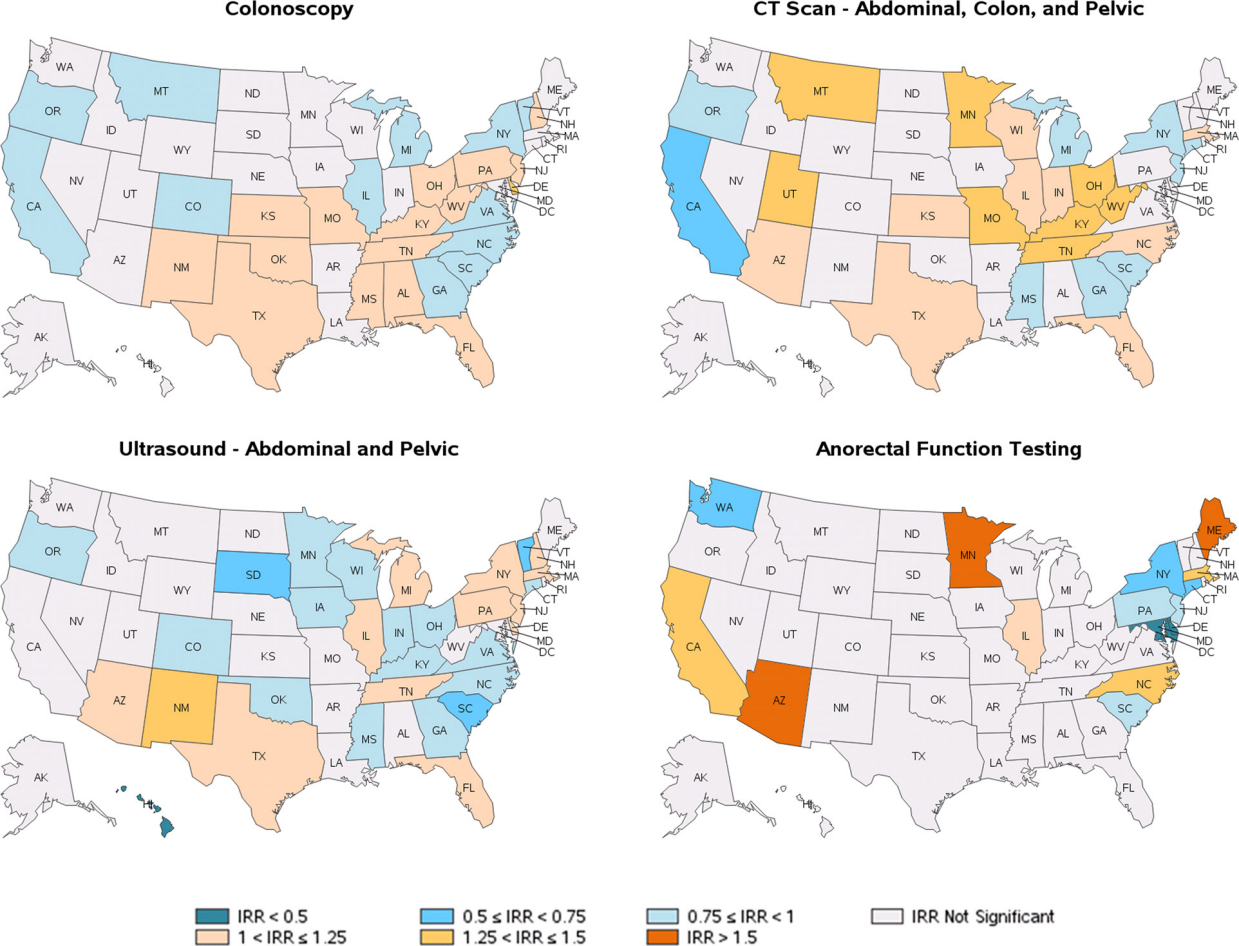
Regional variation of medical procedures and diagnostic tests. Note: For IRR, incidence rate ratio, reference is the rest of US rate. Abbreviations: CT: computed tomography; US, United States. Map data reprinted from SAS software version 9.3 (Cary, NC) under a CC BY license, with permission from GfK GeoMarketing (Bruchsal, Baden-Württemberg, Germany), original copyright 2015.

The incidence of colonoscopy was highest in Delaware (IRR = 1.32) and lowest in Vermont (IRR = 0.75) and California (IRR = 0.75) (all p<0.05) (Fig 1 and Table 3**,** detailing state-specific healthcare resource utilization versus the national average).

**Table 3:** Average Number of Events per Patients.

			Number of Events per Patient (mean ±SD)							
	IBS Sample		Anorectal Function Testing	Colonoscopy	CT Scan—Abdominal, Colon, and Pelvic	Ultrasound—Abdominal and Pelvic	Pharmacy Prescriptions for Treating Constipation or Diarrhea	IP Admissions	ER Visits	GI Specialist Visits
	*N*	%								
***United States***	***201*,*322***	***100%***	***0*.*05 ± 0*.*44***	***0*.*78 ± 1*.*08***	***1*.*33 ± 2*.*84***	***0*.*72 ± 1*.*37***	***0*.*76 ± 2*.*84***	***0*.*09 ± 0*.*39***	***0*.*09 ± 0*.*40***	***1*.*89 ± 2*.*85***
California	25,995	12.9%	0.06 ± 0.44	0.61 ± 0.97	0.88 ± 2.15	0.71 ± 1.39	0.58 ± 2.44	0.07 ± 0.36	0.07 ± 0.37	1.08 ± 2.54
Texas	20,050	10.0%	0.04 ± 0.47	0.90 ± 1.16	1.60 ± 3.50	0.78 ± 1.37	0.66 ± 2.58	0.10 ± 0.41	0.10 ± 0.53	2.57 ± 3.14
Michigan	15,052	7.5%	0.05 ± 0.59	0.71 ± 0.93	1.30 ± 2.64	0.78 ± 1.37	1.33 ± 3.78	0.10 ± 0.40	0.08 ± 0.35	1.58 ± 2.62
Illinois	13,300	6.6%	0.05 ± 0.45	0.79 ± 1.12	1.63 ± 3.23	0.82 ± 1.69	0.52 ± 2.55	0.11 ± 0.42	0.09 ± 0.35	1.51 ± 2.51
Georgia	12,207	6.1%	0.05 ± 0.33	0.66 ± 0.90	1.14 ± 2.45	0.61 ± 1.10	0.75 ± 2.52	0.08 ± 0.36	0.07 ± 0.33	2.75 ± 3.23
South Carolina	11,406	5.7%	0.03 ± 0.28	0.76 ± 1.05	1.21 ± 2.64	0.53 ± 1.05	0.47 ± 2.18	0.08 ± 0.35	0.06 ± 0.31	1.89 ± 2.58
Florida	9,464	4.7%	0.04 ± 0.35	0.91 ± 1.12	1.45 ± 2.89	0.80 ± 1.40	0.69 ± 2.69	0.11 ± 0.41	0.08 ± 0.40	2.49 ± 3.30
Ohio	9,301	4.6%	0.05 ± 0.39	0.80 ± 1.09	1.59 ± 2.96	0.64 ± 1.23	0.96 ± 3.28	0.10 ± 0.40	0.12 ± 0.50	1.79 ± 2.63
New York	9,189	4.6%	0.03 ± 0.43	0.77 ± 1.09	1.08 ± 2.36	0.93 ± 1.68	0.47 ± 2.27	0.07 ± 0.33	0.10 ± 0.38	2.70 ± 3.29
Tennessee	7,096	3.5%	0.05 ± 0.31	0.83 ± 1.10	1.46 ± 3.26	0.76 ± 1.30	1.06 ± 3.15	0.07 ± 0.33	0.07 ± 0.32	2.36 ± 2.86
Indiana	5,758	2.9%	0.04 ± 0.34	0.78 ± 1.15	1.58 ± 2.93	0.55 ± 1.10	1.02 ± 3.29	0.10 ± 0.37	0.10 ± 0.40	1.54 ± 2.55
Pennsylvania	4,888	2.4%	0.04 ± 0.37	0.88 ± 1.21	1.37 ± 2.82	0.80 ± 1.67	0.72 ± 3.01	0.10 ± 0.39	0.09 ± 0.37	1.76 ± 2.66
Missouri	4,625	2.3%	0.04 ± 0.45	0.82 ± 1.21	1.69 ± 3.33	0.72 ± 1.27	0.88 ± 3.17	0.13 ± 0.47	0.13 ± 0.46	1.77 ± 2.74
New Jersey	4,432	2.2%	0.04 ± 0.40	0.95 ± 1.15	1.13 ± 2.65	0.75 ± 1.37	0.72 ± 2.71	0.09 ± 0.37	0.09 ± 0.38	2.72 ± 3.40
North Carolina	3,716	1.8%	0.06 ± 0.92	0.70 ± 1.01	1.47 ± 3.08	0.64 ± 1.53	0.92 ± 3.16	0.08 ± 0.33	0.10 ± 0.40	2.15 ± 3.05
Oklahoma	3,562	1.8%	0.03 ± 0.27	0.85 ± 1.12	1.41 ± 2.72	0.60 ± 1.16	0.60 ± 2.58	0.10 ± 0.41	0.14 ± 0.52	1.60 ± 2.35
Mississippi	3,022	1.5%	0.04 ± 0.24	0.88 ± 1.11	1.04 ± 2.34	0.59 ± 1.09	1.07 ± 2.97	0.11 ± 0.41	0.08 ± 0.37	0.88 ± 2.18
Alabama	2,832	1.4%	0.03 ± 0.24	0.94 ± 1.11	1.42 ± 3.23	0.74 ± 1.31	1.01 ± 3.12	0.10 ± 0.39	0.10 ± 0.44	1.90 ± 2.64
Kentucky	2,808	1.4%	0.06 ± 0.92	0.92 ± 1.23	1.77 ± 3.20	0.63 ± 1.16	1.13 ± 3.73	0.12 ± 0.46	0.11 ± 0.41	1.62 ± 2.51
Massachusetts	2,594	1.3%	0.07 ± 0.42	0.78 ± 1.03	1.25 ± 2.60	0.85 ± 1.55	0.60 ± 2.44	0.08 ± 0.40	0.09 ± 0.41	1.85 ± 2.62
Washington	2,558	1.3%	0.03 ± 0.26	0.82 ± 1.12	1.20 ± 2.58	0.71 ± 1.48	0.70 ± 2.72	0.06 ± 0.35	0.06 ± 0.31	1.86 ± 2.80
Virginia	2,327	1.2%	0.04 ± 0.28	0.69 ± 0.98	1.35 ± 2.76	0.62 ± 1.27	1.10 ± 3.55	0.10 ± 0.43	0.11 ± 0.46	2.02 ± 2.83
Connecticut	2,287	1.1%	0.02 ± 0.23	0.83 ± 1.16	1.07 ± 2.33	0.64 ± 1.47	0.68 ± 2.81	0.07 ± 0.31	0.09 ± 0.35	1.77 ± 2.78
Arizona	2,082	1.0%	0.08 ± 0.63	0.77 ± 1.11	1.45 ± 3.41	0.79 ± 1.58	0.77 ± 2.78	0.11 ± 0.41	0.09 ± 0.37	1.79 ± 2.77
New Mexico	1,806	0.9%	0.04 ± 0.33	0.88 ± 1.24	1.49 ± 3.02	0.98 ± 1.56	0.31 ± 1.70	0.09 ± 0.38	0.09 ± 0.37	1.65 ± 2.36
Wisconsin	1,697	0.8%	0.04 ± 0.38	0.78 ± 1.08	1.40 ± 2.80	0.63 ± 1.20	0.74 ± 2.82	0.10 ± 0.38	0.11 ± 0.43	1.39 ± 2.39
Maryland	1,598	0.8%	0.02 ± 0.29	0.82 ± 1.06	1.41 ± 3.11	0.70 ± 1.22	0.85 ± 3.01	0.10 ± 0.46	0.10 ± 0.38	2.22 ± 2.88
Oregon	1,496	0.7%	0.03 ± 0.28	0.71 ± 1.04	1.07 ± 2.39	0.68 ± 1.34	0.38 ± 2.06	0.06 ± 0.30	0.06 ± 0.28	1.02 ± 2.01
Nevada	1,472	0.7%	0.04 ± 0.26	0.80 ± 1.15	1.22 ± 2.51	0.64 ± 1.13	0.67 ± 2.60	0.08 ± 0.36	0.08 ± 0.38	1.99 ± 2.83
Colorado	1,419	0.7%	0.05 ± 0.49	0.67 ± 0.94	1.28 ± 2.59	0.54 ± 1.02	0.74 ± 2.90	0.09 ± 0.34	0.12 ± 0.43	1.78 ± 2.61
Kansas	1,355	0.7%	0.04 ± 0.30	0.87 ± 1.09	1.44 ± 3.06	0.72 ± 1.42	1.09 ± 3.54	0.10 ± 0.43	0.09 ± 0.36	1.30 ± 2.55
Louisiana	1,275	0.6%	0.04 ± 0.28	0.80 ± 1.02	1.38 ± 2.64	0.79 ± 1.42	1.13 ± 3.85	0.14 ± 0.49	0.07 ± 0.30	1.83 ± 2.76
Arkansas	1,165	0.6%	0.05 ± 0.43	0.84 ± 1.06	1.39 ± 2.68	0.64 ± 1.11	0.96 ± 3.04	0.10 ± 0.35	0.09 ± 0.37	1.54 ± 2.57
Delaware	1,117	0.6%	0.01 ± 0.18	1.03 ± 1.16	1.33 ± 2.70	0.86 ± 1.40	0.75 ± 2.87	0.07 ± 0.35	0.05 ± 0.27	2.50 ± 2.84
Iowa	996	0.5%	0.05 ± 0.36	0.75 ± 1.03	1.33 ± 2.67	0.61 ± 1.17	0.79 ± 2.76	0.12 ± 0.44	0.10 ± 0.39	1.72 ± 2.81
West Virginia	910	0.5%	0.05 ± 0.27	0.93 ± 1.17	1.86 ± 3.51	0.81 ± 1.41	0.99 ± 3.42	0.12 ± 0.39	0.11 ± 0.38	1.61 ± 2.88
New Hampshire	683	0.3%	0.06 ± 0.36	0.91 ± 1.28	1.33 ± 2.72	0.93 ± 1.57	0.79 ± 3.11	0.10 ± 0.34	0.16 ± 0.91	1.78 ± 2.88
Maine	638	0.3%	0.08 ± 0.48	0.86 ± 1.39	1.27 ± 2.36	0.70 ± 1.27	0.63 ± 2.70	0.08 ± 0.33	0.13 ± 0.42	0.99 ± 2.20
Minnesota	602	0.3%	0.17 ± 0.96	0.66 ± 0.97	1.60 ± 3.19	0.64 ± 1.16	0.78 ± 2.65	0.11 ± 0.36	0.16 ± 0.60	1.06 ± 2.37
Montana	526	0.3%	0.05 ± 0.34	0.68 ± 0.99	1.89 ± 3.52	0.62 ± 1.32	0.27 ± 1.57	0.18 ± 0.52	0.12 ± 0.42	1.23 ± 2.33
Nebraska	434	0.2%	0.04 ± 0.33	0.74 ± 0.96	1.40 ± 2.89	0.68 ± 1.26	0.88 ± 3.06	0.09 ± 0.38	0.06 ± 0.26	1.07 ± 2.09
Utah	361	0.2%	0.04 ± 0.31	0.70 ± 0.99	1.59 ± 3.45	0.70 ± 1.57	0.61 ± 2.18	0.09 ± 0.37	0.12 ± 0.41	1.22 ± 2.72
Rhode Island	353	0.2%	0.05 ± 0.56	0.83 ± 1.18	1.35 ± 2.66	0.79 ± 1.29	0.78 ± 2.45	0.05 ± 0.28	0.07 ± 0.28	2.57 ± 3.39
Idaho	261	0.1%	0.02 ± 0.14	0.83 ± 1.09	1.34 ± 2.91	0.65 ± 1.34	0.59 ± 2.25	0.07 ± 0.33	0.13 ± 0.47	1.60 ± 2.92
Vermont	145	0.1%	0.06 ± 0.39	0.57 ± 0.78	1.02 ± 3.03	0.46 ± 0.96	1.02 ± 3.08	0.08 ± 0.36	0.10 ± 0.32	0.56 ± 1.24
South Dakota	130	0.1%	0.05 ± 0.27	0.79 ± 1.94	1.22 ± 2.43	0.48 ± 1.00	0.87 ± 2.74	0.09 ± 0.32	0.12 ± 0.41	1.60 ± 3.80
Alaska	113	0.1%	0.04 ± 0.28	0.72 ± 0.93	1.08 ± 2.41	0.60 ± 1.15	0.37 ± 1.23	0.04 ± 0.31	0.12 ± 0.52	0.92 ± 1.97
Wyoming	87	0.04%	0.03 ± 0.24	0.86 ± 1.21	1.82 ± 2.79	0.61 ± 1.21	0.70 ± 2.53	0.14 ± 0.38	0.07 ± 0.25	0.61 ± 1.30
North Dakota	76	0.04%	0.04 ± 0.34	0.72 ± 1.05	1.45 ± 2.75	0.96 ± 1.64	0.07 ± 0.47	0.16 ± 0.46	0.11 ± 0.35	1.01 ± 2.54
Washington, DC	36	0.02%	0.00 ± 0.00	0.75 ± 1.00	1.83 ± 3.70	0.61 ± 1.05	0.41 ± 1.24	0.08 ± 0.28	0.19 ± 0.47	2.97 ± 4.20
Hawaii	20	0.01%	0.00 ± 0.00	0.50 ± 0.69	0.85 ± 1.27	0.25 ± 0.55	2.64 ± 6.26	0.00 ± 0.00	0.05 ± 0.22	1.55 ± 2.65

Abbreviations: IBS: irritable bowel syndrome; CIs: confidence intervals. CT: computed tomography; ER: emergency room; GI: gastrointestinal; IP: inpatient; SD, standard deviation; DC: District of Columbia.

A little more than one-third (34.6%) of all IBS patients had CT scans, averaging 1.33 CT scans per patient (Table 2). Significant differences were observed in CT scan utilization: Kentucky, Missouri, Montana, Minnesota, West Virginia, Tennessee, Utah, and Ohio had higher incidences than the rest of the country (IRRs = 1.27 to 1.36; all p<0.05), while California (IRR = 0.63; p<0.001) had the lowest incidence (Fig 1 and Table 3, detailing state-specific healthcare resource utilization versus the national average).

Abdominal and pelvic ultrasounds were conducted in 35.2% of the IBS patients, with an average of 0.72 ultrasounds per patient (Table 2). They were most frequent in New Mexico (IRR = 1.26; p<0.001) and least in Hawaii (IRR = 0.33; p = 0.042) (Fig 1 and Table 3).

Anorectal function testing was conducted in 2.7% of the IBS patients with an overall average of 0.05 tests per patient (Table 2.A). Anorectal function testing was most frequent in Minnesota (IRR = 4.05), Maine (IRR = 2.25), and Arizona (IRR = 1.50) and least frequent in Delaware (IRR = 0.32) and Maryland (IRR = 0.49) (all p<0.05) (Fig 1).

**Fig 2:**
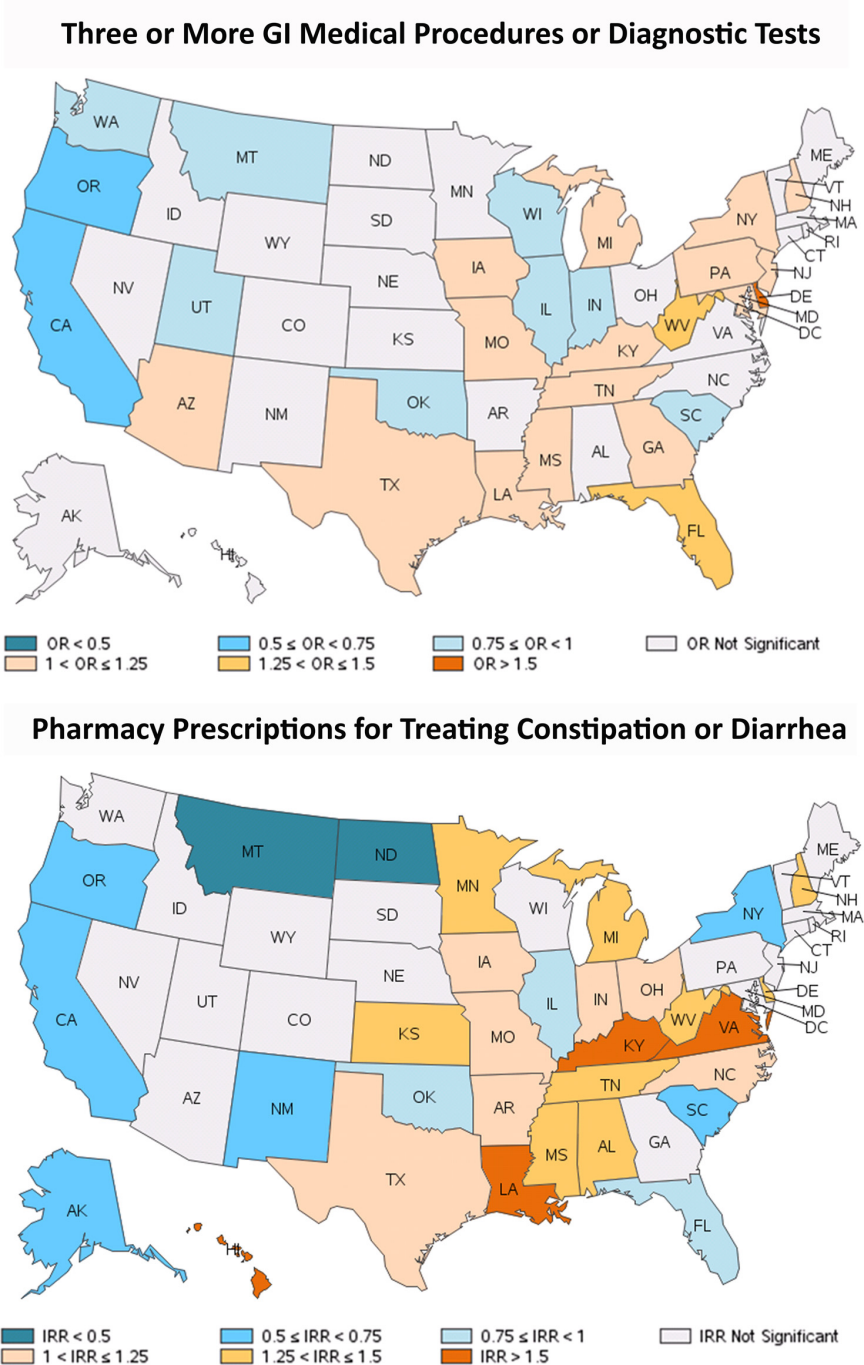
Regional variation of GI medical procedures or diagnostic tests and pharmacy prescriptions for treating constipation or diarrhea. Note: For OR, odds ratio, reference is the rest of US rate. Abbreviations: GI: gastrointestinal; US, United States. Map data reprinted from SAS software version 9.3 (Cary, NC) under a CC BY license, with permission from GfK GeoMarketing (Bruchsal, Baden-Württemberg, Germany), original copyright 2015.

A sizable proportion of patients (36.3%) had three or more different types of GI medical procedures or diagnostic tests during the study period (Table 2). Patients in Delaware, Florida, and West Virginia were the most likely to receive three or more different types of procedures or tests (ORs: 1.32 to 1.70), while patients from California and Oregon were the least likely (ORs: 0.65 and 0.65) (all p<0.001) (Fig 2).

One-third of patients had prescription pharmacy claims for constipation or diarrhea treatments. Overall, 29.0% were prescribed an anti-constipation medication, and 7.5% an anti-diarrheal medication (Table 2). The use of medications for constipation or diarrhea was particularly high in the Southern and Central regions of the US (Fig 2 and Table 3).

**Fig 3:**
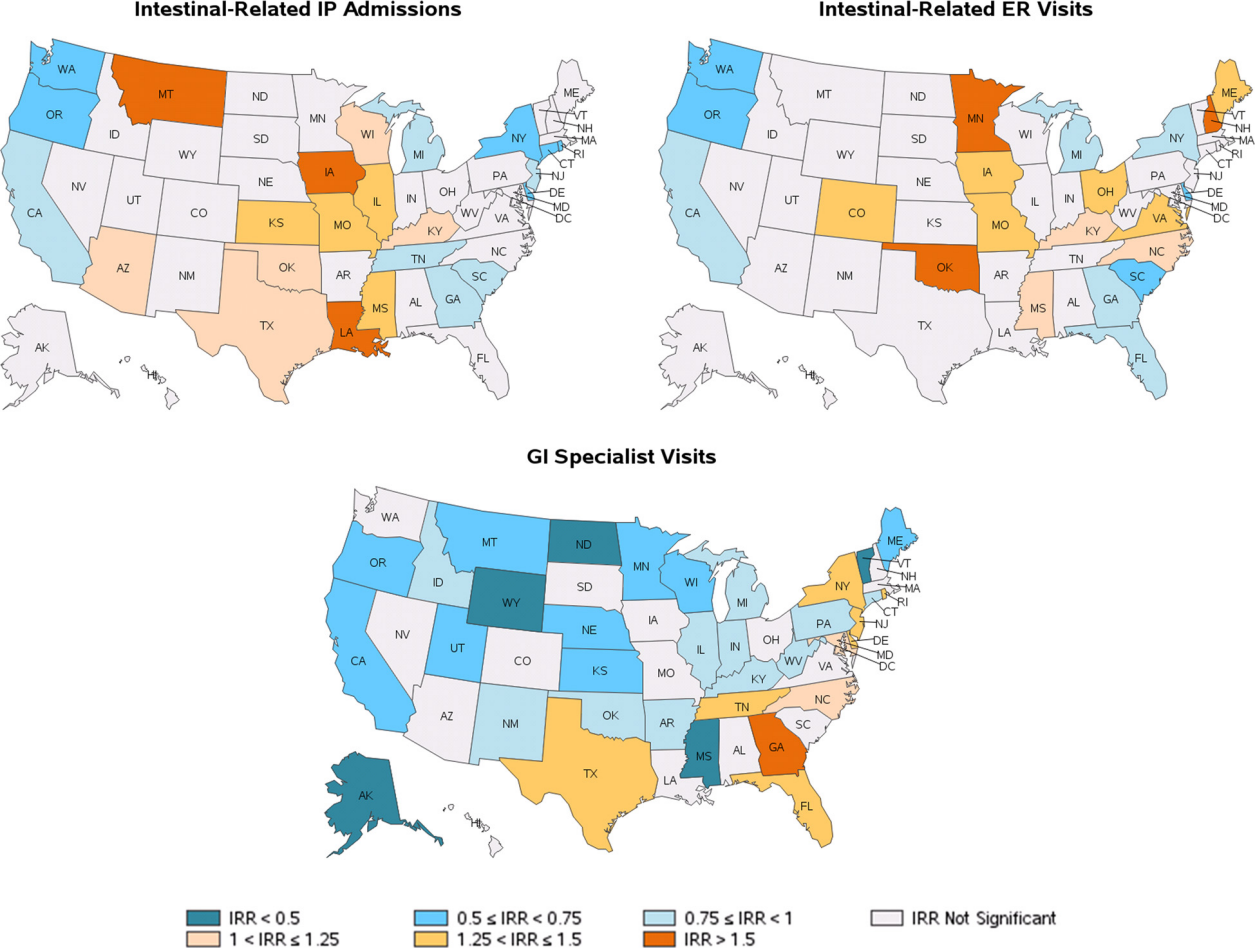
Regional variation of the frequency of intestinal-related IP admissions, ER visits, and GI specialist visits. Note: For IRR, incidence rate ratio, reference is the rest of US rate. Abbreviations: ER: emergency room; GI: gastrointestinal; IP: inpatient; US, United States. Map data reprinted from SAS software version 9.3 (Cary, NC) under a CC BY license, with permission from GfK GeoMarketing (Bruchsal, Baden-Württemberg, Germany), original copyright 2015.

Regarding medical visits, 7.3% of patients had at least one intestinal-related IP admission, with an average of 0.09 IP admissions over the study period (Table 2). Rates of IP admissions were highest in Montana, Iowa, and Louisiana (IRRs = 1.55 to 2.05) and lowest in Rhode Island, Oregon, New York, Washington, and Delaware (IRRs = 0.52 to 0.73) (all p<0.05) (Fig 3 and Table 3). In addition, 6.8% of the IBS patients had at least one intestinal-related ER visit, with an average of 0.09 ER visits per patient (Table 2 and Table 3). These were most frequent in Minnesota, New Hampshire, and Oklahoma (IRRs = 1.58 to 1.89) and least frequent in Delaware, Oregon, South Carolina, and Washington (IRRs = 0.54 to 0.75) (all p<0.05) (Fig 3 and Table 3).

Half of patients visited a GI specialist during the study period, for an average of 1.89 GI specialist visits per patient (Table 2). Significant differences were observed in GI specialist visits: Georgia had the highest incidence (IRR = 1.66), while Wyoming, Vermont, Mississippi, Alaska and North Dakota had the lowest (IRRs = 0.30 to 0.48) (all p<0.001) (Fig 3 and Table 3).

Compared to IBS patients without constipation, IBS-C patients were found to have higher HRU in all categories, except for anti-diarrheal medications, where IBS patients without constipation had higher use of anti-diarrheal medications. (results stratified for pharmacy prescriptions for treating constipation and diarrhea separately not presented). IBS-C patients had 42% more colonoscopies, 63% more CT scans, 35% more ultrasounds, and more than 4 times as much anorectal function testing compared to IBS patients without constipation. IBS-C patients also had more than 3 times more intestinal-related ER visits, 91% more inpatient admissions, and 55% more GI specialist visits (Table 4).

**Table 4:** Comparison of HRU between IBS-C patients and IBS patients without constipation.

	Unadjusted IRRs^a^ (95% CIs)	Adjusted IRRs^a^ (95% CIs)
**Medical Procedures and Diagnostic Tests**		
Colonoscopy	1.43 (1.41–1.45)	1.42 (1.40–1.44)
CT scan	1.80 (1.75–1.84)	1.63 (1.59–1.67)
Ultrasound	1.50 (1.47–1.53)	1.35 (1.32–1.38)
Anorectal Function Testing	3.67 (3.41–3.96)	4.14 (3.84–4.46)
**Pharmacy prescriptions for treating constipation or diarrhea**	3.27 (3.17–3.38)	3.42 (3.31–3.53)
**Medical Visits**		
Intestinal-Related IP Admissions	2.19 (2.10–2.28)	1.91 (1.84–1.99)
Intestinal-Related ER Visits	3.55 (3.42–3.69)	3.19 (3.08–3.32)
GI Specialist Visits	1.61 (1.58–1.64)	1.55 (1.52–1.57)

Note:

^a^ An IRR > 1 indicates that IBS-C patients had higher HRU compared to IBS patients without constipation; while an IRR < 1 indicates that IBS-C patients had lower HRU compared to IBS patients without constipation. All p<0.001.

Abbreviations: CIs: confidence intervals. CT: computed tomography; ER: emergency room; GI: gastrointestinal; HRU, healthcare resource utilization; IBS, irritable bowel syndrome; IBS-C, irritable bowel syndrome with constipation; IP: inpatient; IRR: incidence rate ratio.

## Discussion

To our knowledge, this is the first study to analyze regional variation patterns in IBS care in the US. IBS is a highly prevalent disease [1,33] and its associated costs are substantial [34,35]. As a mechanism of lowering existing costs, an investigation of treatment and diagnostic efficacy or variation in care is warranted, as suboptimal and inefficient use of services generally dramatically increase costs. A better understanding of HRU and of variations in healthcare will enable payers and policy makers to identify opportunities for standardization in suitable areas, which, in turn, should improve the quality of patient care and reduce the economic burden on payers and society.

We identified a number of significant regional variations in IBS care. Approximately 36% of patients underwent three or more distinct types of GI medical procedures or diagnostic tests during the 24-month study period before and after their IBS diagnosis. Many had colonoscopies and CT scans. Current clinical guidelines recommend that IBS be diagnosed based on a careful clinical history and examination, in the absence of warning signs. There is little consensus on the use of imaging tools and tests in the diagnosis and treatment of IBS [8,36]. For example, abdominal ultrasounds have been shown to have very little or no value for the diagnosis or management of IBS [36]; however, 35.2% of patients in our study sample had at least one abdominal or pelvic ultrasound. Although ultrasounds could have been performed for reasons unrelated to IBS, the high rate of use suggests the possibility of unnecessary use in a proportion of patients, especially given that many were performed in men. Overall, California showed generally lower use of imaging tests than the national average, including the lowest likelihood for IBS patients to undergo three or more different types of GI medical procedures or tests, though the underlying reasons for this observation remain elusive. Similarly, significant variations were observed in intestinal-related IP admissions, ER visits, and GI specialist visits. No specific patterns were discerned. Pharmacy prescriptions for treating constipation or diarrhea appeared to be higher in the Southern and Central regions of the US and lower in the Western and Northern regions.

The underlying reasons for the geographic variations in IBS care are likely multiple and may be difficult to fully elucidate. Potential influential factors of regional variations in IBS care include differences in population characteristics and comorbidities, insurance plan types, types of coverage, patterns of medical practice, fear of being sued for medical malpractice, availability of GI specialists, patterns of medical education and training, and clustering of care in academic or tertiary-care centers. In this study we attempted to account for some of these variable factors across states in the multivariate regression models. For example, the type of insurance plan was noted to impact regional variation in care. Specifically, we found that patients with Health Maintenance Organization (HMO) insurance had fewer GI specialist visits, IBS-related pharmacy prescriptions, colonoscopies and CT scans, but more ultrasound and anorectal function testing compared to patients with Preferred Provider Organization (PPO) insurance (data not shown). In the states with a lower average number of GI specialists per capita than the national average [37], we found significantly fewer to similar numbers of GI specialist visits compared with the rest of the US. This suggests that the number of GI specialist visits is likely to be associated with the accessibility of such specialists. Using the data from National Practitioners Data Bank (NPDB) [38], we found no clear trend between the number of malpractice cases per state per capita and the IRR of procedures or tests per state. Further analyses are needed to identify the reasons for the regional variations.

In addition to variations by state, we investigated certain urban areas that are home to major academic or clinical centers, such as Rochester and St. Paul in Minnesota, Jacksonville in Florida, and Scottsdale and Tucson in Arizona, to better understand variations in diagnostic testing. Anorectal manometry was chosen for this analysis because it is a diagnostic test that may identify the etiology of constipation or incontinence in patients with IBS-C and IBS-D, respectively. The results suggested that all the mentioned metro areas above, except Scottsdale, had a significantly higher utilization of anorectal function testing than the rest of their respective states (data not shown). Further analysis and comparison of HRU among cities in the same state showed that higher utilization does not necessarily correlate with the size of the cities, but rather with the existence of major healthcare centers in those cities. For instance, in Florida, Jacksonville had significantly higher anorectal function testing utilization than the rest of the state, but Miami, Orlando, and Tampa did not show higher rates of anorectal function testing.

We also compared HRU between IBS-C patients and IBS patients without constipation. We chose to focus on IBS-C because previous evidence suggested high healthcare costs associated with this subtype [30,39,40]. Our analysis confirms that IBS-C patients had significantly more GI medical procedures and diagnostic tests, more prescription fills, and more IP admissions, ER visits, and GI specialist visits than IBS patients without constipation. These findings suggest that the IBS-C subtype may be a potential target for cost-effective interventions.

The observed substantial regional variations in IBS care highlight the need for more evidence-based diagnosis and treatment guidelines, based on large population studies, so that clinicians can provide more consistent care on a national level. Although our analyses hint at several causes of inconsistencies in IBS care, further research is needed to more precisely determine these causes, in order to promote optimal and efficient healthcare services across the country. Improving the dissemination and adoption of best practices in IBS care has the potential to reduce the economic burden of IBS on a national level.

This study has several limitations. First, the analysis was performed using a commercially insured population, which may not be fully representative of all US patients in all age ranges. Second, the period for analysis was 24 months, which may not necessarily capture long-term trends and patterns of IBS care. Third, the multivariate regression analyses were adjusted for confounders such as age, gender, comorbidity index, insurance plan type, and the year of first IBS diagnosis. Different values of each of these confounders introduce a class of patients sharing the same value for that confounder. An inherent limitation in regression analysis is that it does not identify or take into account the variations that may exist within each of these classes of patients. In addition, other confounders may remain unadjusted for. That is, the patient sample may have different unmeasured characteristics that multivariable regression analyses did not account for. For example, race data were not available from the datasets. Fourth, data on over-the-counter medication use was not available in the database. Fifth, there is no specific ICD-9 code that identifies IBS subgroups (i.e., IBS-C). Therefore, patients were first classified into an IBS subtype by using the standard ICD-9 code for IBS and then sub-classified using concomitant codes for either constipation or diarrhea. However, since some IBS patients change subtype over time, it is possible that a patient with one IBS subtype (i.e., IBS-M) could have been misclassified as another subtype (i.e. IBS-C). As patients can change subgroups in a bidirectional manner, however, the effect of this should be minimal. Finally, because of space limitations, we could not report all analyses conducted on the whole host of HRU measures. Nevertheless, the omitted results are generally consistent with those we have presented and confirm the regional variations in health care provided to IBS patients.

To conclude, this large population-based study of IBS patients showed considerable regional variations of care across the US and substantially higher use of healthcare resources by IBS-C patients than by IBS patients without constipation. Identifying the reasons for these variations may improve quality of care and reduce the economic burden of IBS care.

## Supporting Information

S1 Table Pharmacy prescriptions for treating constipation or diarrhea. (DOCX)
