# Supplementary material for: Variation in Care for Patients with Irritable Bowel Syndrome in the United States
Source: PLoS One. 2016 Apr 26;11(4):e0154258. doi: 10.1371/journal.pone.0154258 (PMC4845999; doi:10.1371/journal.pone.0154258)
Supplement: S1 Table — (DOCX) [file pone.0154258.s001.docx]

# Supplementary Material

**S1 Table. Pharmacy prescriptions for treating constipation or diarrhea**

| **Pharmacy Prescriptions for Treating Constipation** | **Pharmacy Prescriptions for Treating Diarrhea** |
| --- | --- |
| **Laxatives, Osmotic** | Diphenoxylate |
| Polyethylene Glycol (PEG) | Loperamide |
| Visicol | Miscellaneous Intestinal Flora Regulator |
| Lactulose | Paregoric |
| Sodium Phosphate | Opium Tincture |
| Magnesium | Difenoxin |
| Magnesium Citrate | Lactobacillus |
| Sorbitol | Bismuth |
| Prepopik | Saccharomyces |
| **Laxatives, Stimulant** | Probiotic Product |
| Bisacodyl |  |
| Sennosides |  |
| Sennosides Docusate Sodium |  |
| Phenolphthalein |  |
| Cascara |  |
| Castor Oil |  |
| **Bulk-Forming Agent** |  |
| Psyllium |  |
| Calcium Polycarbophil |  |
| Methylcellulose |  |
| Cellulose |  |
| Inulin |  |
| Wheat Dextrin |  |
| Corn Dextrin Powder |  |
| **Stool Softener** |  |
| Docusate Sodium |  |
| Poloxamer |  |
| **Other Prescription Medications Indicated for Irritable Bowel Syndrome (IBS)** |  |
| Tegaserod |  |
| Lubiprostone |  |
| Linaclotide |  |
